# Antitumor Cannabinoid Chemotypes: Structural Insights

**DOI:** 10.3389/fphar.2019.00621

**Published:** 2019-05-31

**Authors:** Paula Morales, Nadine Jagerovic

**Affiliations:** Instituto de Quimica Medica, Consejo Superior de Investigaciones Cientificas, Madrid, Spain

**Keywords:** cannabinoid, cancer, ADMET, in silico, chemotype

## Abstract

Cannabis has long been known to limit or prevent nausea and vomiting, lack of appetite, and pain. For this reason, cannabinoids have been successfully used in the treatment of some of the unwanted side effects caused by cancer chemotherapy. Besides their palliative effects, research from the past two decades has demonstrated their promising potential as antitumor agents in a wide variety of tumors. Cannabinoids of endogenous, phytogenic, and synthetic nature have been shown to impact the proliferation of cancer through the modulation of different proteins involved in the endocannabinoid system such as the G protein–coupled receptors CB1, CB2, and GRP55, the ionotropic receptor TRPV1, or the fatty acid amide hydrolase (FAAH). In this article, we aim to structurally classify the antitumor cannabinoid chemotypes described so far according to their targets and types of cancer. In a drug discovery approach, their *in silico* pharmacokinetic profile has been evaluated in order to identify appropriate drug-like profiles, which should be taken into account for further progress toward the clinic. This analysis may provide structural insights into the selection of specific cannabinoid scaffolds for the development of antitumor drugs for the treatment of particular types of cancer.

## Introduction

During these last years, significant research has been focused on the therapeutic potential of cannabinoids to manage palliative effects in cancer patients (Badowski, 2017; Guzmán, 2018). Cancer-induced emesis represents the initial target indication for Marinol^®^ (dronabinol) and Cesamet^®^ (nabilone), two cannabis-based medicines approved by various regulatory drug agencies. Management of cancer-induced neuropathic pain is also part of the palliative applications of cannabis-based medicines. Besides such palliative applications, some cannabinoids have shown anticancer properties (Guzmán, 2003; Guindon and Hohmann, 2011; Khan et al., 2016; Hinz and Ramer, 2019). As widely reported in the last decades, some cannabinoids are able to modulate different cellular signaling pathways implicated in cancer cell proliferation, migration, or death (Chakravarti et al., 2014). Even though the underlying mechanisms are not totally unraveled, there is significant evidence for the involvement of at least four mechanisms: direct inhibition of transformed-cell growth through the suppression of mitogenic signal, induction of apoptosis, inhibition of tumor angiogenesis, and metastasis (Velasco et al., 2016). The signaling pathways implicated in the activation of the endocannabinoid system may differ depending on specific cancers and/or experimental models, making more complex the understanding of these processes. So far, only few clinical data on the efficacy of cannabinoids as anticancer agents have been provided (Ladin et al., 2016). However, great efforts are currently being made to elucidate the potential utility of cannabinoids as anticancer therapeutics.

The physiological processes triggered by most of these cannabinoids are mediated by two G protein–coupled cannabinoid receptors (CBR), CB1R and CB2R. CB1R is predominantly and abundantly expressed in the central nervous system, with predominance in the hippocampus, cerebellum, basal ganglia, and cortical and olfactory regions, but CB1R is also present in many organs of the peripheral system. CB2R is mainly found in the immune system, in the spleen, thymus, lymph nodes, and tonsils, but it is also expressed in immune cells.

The overexpression of CBR and elevated endocannabinoid levels have been reported in different cancer types (Blázquez et al., 2006; Pisanti et al., 2013). This expression in cancer cells is crucial for downstream signaling with implications on cell viability.

Non-CB1R, non-CB2R targets related to the endocannabinoid system have also been reported to be involved in the anticancer action of cannabinoids. For instance, specific effects may be due to interactions with enzymes of the endocannabinoid system such as FAAH (fatty acid amide hydrolase), NAPE-PLD (*N*-acyl phosphatidylethanolamine phospholipase D), MAGL (monoacylglycerol lipase), DAGL (diacylglycerol lipase), ABHD6 (α/β-hydrolase domain containing 6), or ABHD12 (α/β-hydrolase domain containing 12); with GPR55 and/or GPR18, two putative cannabinoid orphan G protein–coupled receptors; with transient receptor potential (TRP) channels (TRPV1–4, TRPM8, and TRPA1); or with COX-2 (cyclooxygenase-2), among others (Morales et al., 2017a; Morales and Reggio, 2017).

Focusing on a drug discovery approach, herein we have summarized the anticancer profiling of the cannabinoids reported thus far to impact cancer. Moreover, we have calculated their *in silico* pharmacokinetic profiles in order to predict appropriate drug-like profiles that may provide useful criteria for further development selection. *In silico* prediction of pharmacokinetic properties is a very useful approach that provides a great translational tool since absorption, distribution, metabolism, excretion, and toxicity (ADMET) properties and bioavailability of drugs can strongly influence their development (Di et al., 2018).

## Cannabinoids with Anticancer Potential

Molecules that modulate the endocannabinoid system are considered cannabinoids. These compounds generally have been classified following their structural nature or origin. Thus, they all belong to phytogenic-, endogenous-, or synthetic-derived families.

### Endocannabinoids

Endogenous cannabinoids, called endocannabinoids, such as anandamide (AEA) and 2-arachidonoyl glycerol (2-AG), form a major family of cannabinoids (Ligresti et al., 2016). Structurally, they are lipid-based derivatives derived from arachidonic acid. They are involved in a number of physiological processes but are also easily degraded through enzymatic pathways. AEA is known to affect cancer cell proliferation; however, there are cell lines whose proliferation is more sensitive to anandamide than others. The molecular mechanism of action differs also from one cell line to another. For instance, AEA exerts a potent CB1R-mediated effect on the proliferation of MCF-7, and EFM-10 human breast cancer cells (Di Marzo et al., 1998), while in N18TG2 murine neuroblastoma cells, the effect is due to FAAH-mediated degradation of AEA to ethanolamine (Matas et al., 2007). Another example concerns non-melanoma skin cancer, for which AEA induces endoplasmic reticulum stress and apoptosis mediated by oxidative stress and by CBR-independent endocannabinoid signaling (Soliman and Van Dross, 2016).

(*R*)-Methanandamide (Met-AEA, **Table 1**) has been used in diverse biological assays as a metabolic stable anandamide analogue. One of the first assays in cancer concerns prostate LNCaP cells (Sánchez et al., 2003).

**Table 1:** Cannabinoids exerting anticancer effects.

Compound	Targeted tumor	Antitumor effect/mechanism of action	References
*Endocannabinoids and derivatives*			
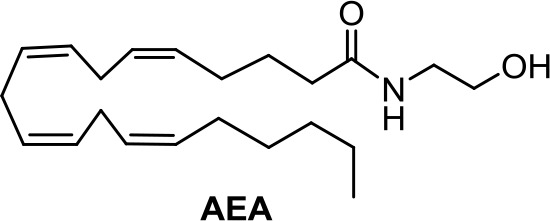	Non-melanoma skin cancer: JWF2 cells	Induces apoptosis mediated by oxidative stress and by CB receptor–independent endocannabinoid signaling	(Soliman and Van Dross, 2016)
	Breast cancer: MCF-7 and EFM-10 cells	Blocks cancer proliferation through CB1R-mediated inhibition of endogenous prolactin action	(Di Marzo et al., 1998)
	Neuroblastoma: N18TG2 cells	Neuroprotection from apoptosis mediated by FAAH	(Matas et al., 2007)
	Prostate cancer: PC-3 cells	Inhibits cancer cell proliferation via CB1R	(Mimeault et al., 2003)
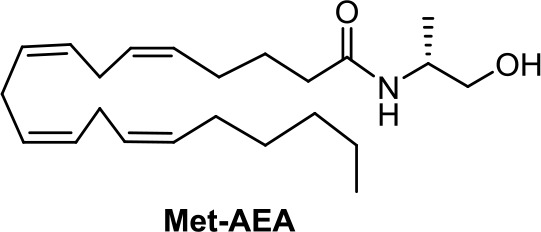	Gastric cancer: human AGS adenocarcinoma cells	Apoptosis induction	(Ortega et al., 2016)
	Prostate cancer: LNCaP	Upregulation of androgen receptor expression	(Sánchez et al., 2003)
	Breast cancer: MDA-MB-231	Inhibition of cell adhesion and migration	(Grimaldi et al., 2006)
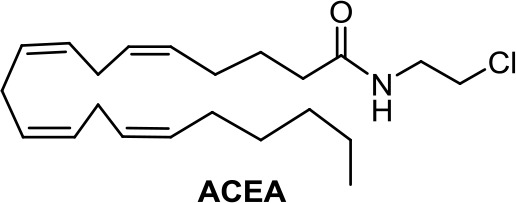	Breast cancer: MDA-MB-231 cells	Decreases cancer stem cell invasiveness	(Mohammadpour et al., 2017)
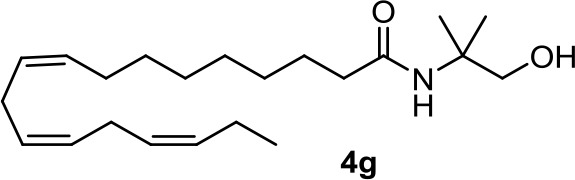	Glioma: C6 cells	Inhibits cell proliferation, enhancing AEA cytotoxicity (via FAAH inhibition)	(Quintana et al., 2016)
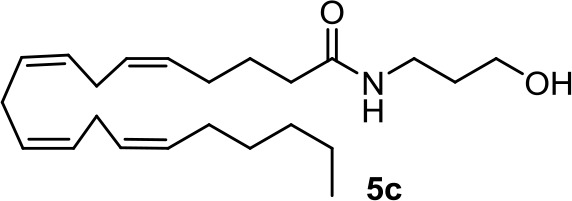	Glioma: C6 cells	Inhibits cell proliferation, enhancing AEA cytotoxicity (via FAAH inhibition)	(Quintana et al., 2016)
*Phytogenic compounds*			
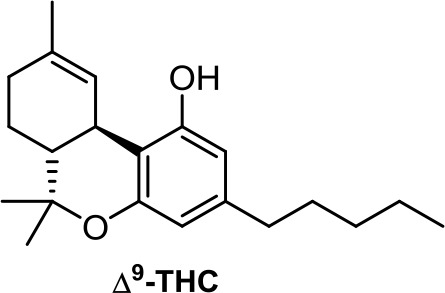	Endometrial cancer: HEC-1B and An3ca cells	Inhibits metastasis by targeting matrix MMP9	(Zhang et al., 2018b)
	Breast cancer: MDA-MB-231, MCF-7	Induces apoptosis	(Ligresti, 2006)
	Prostate cancer: PC-3 cells	Induces cell death and apoptosis	(Ruiz et al., 1999)
	Glioma: human GBM tumor samples	Reduces tumor growth	(Velasco et al., 2016; Dumitru et al., 2018; López-Valero et al., 2018)
	Leukemia: CEM, HEL-92, and HL60 cells	Induces apoptosis	(Powles et al., 2005)
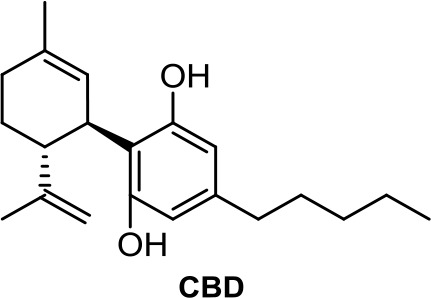	Breast cancer: MDA-MB-231, MCF-7	Induces apoptosis, inhibiting cell viability via CB2R and TRPV1	(Ligresti, 2006)
	Prostate cancer: LNCaP cells	Inhibits cell proliferation and induces apoptosis	(Sreevalsan et al., 2011)
	Glioma: glioma stem cells	Reduces tumor growth	(Singer et al., 2015; Hinz and Ramer, 2019)
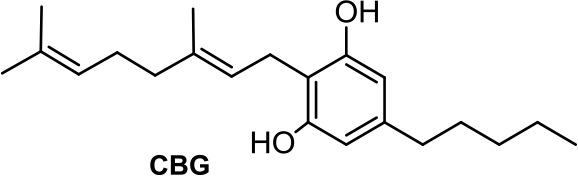	Breast cancer: MDA-MB-231, MCF-7	Induces apoptosis, inhibiting cell viability	(Ligresti, 2006)
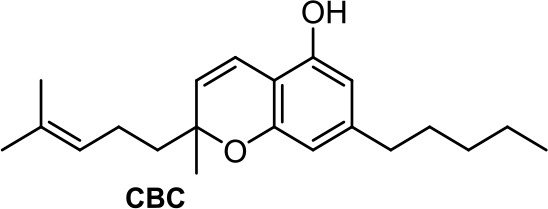	Breast cancer: MDA-MB-231, MCF-7	Induces apoptosis, inhibiting cell viability	(Ligresti, 2006)
	Prostate cancer: DU-145 and LNCaP cells	Inhibits cell proliferation	(De Petrocellis et al., 2013)
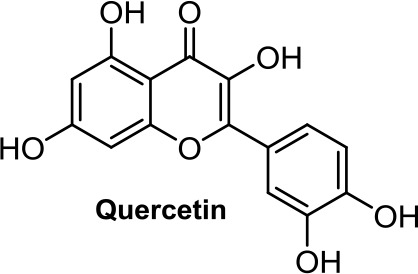	Colon cancer: Caco-2 and DLD-1 cells	Proapoptotic effects mediated through CB1R	(Refolo et al., 2015)
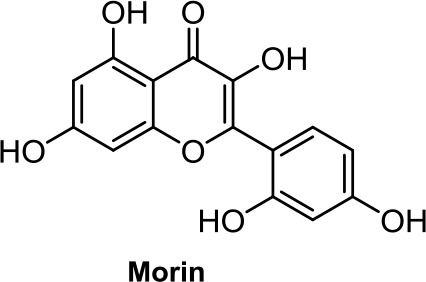	Bone cancer: tibia bone cancer rat model	Suppresses astrocyte activation and neuro-inflammation in bone cancer pain via CB2R activation	(Jiang et al., 2017)
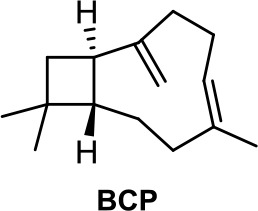	Colon and pancreas cancer: HCT116, HT-29, and PANC-1 cells	Antiproliferative effects	(Dahham et al., 2015)
Synthetic cannabinoids			
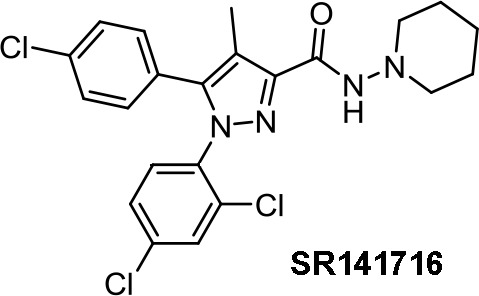	Colon cancer: HCT116 and DLD-1 cells	Reduces both tumor differentiated and cancer stem cell proliferation	(Fiore et al., 2018)
	Colon cancer: HCT116 and SW48 cells	Reduces tumor growth and destabilizes the nuclear localization of β-catenin	(Proto et al., 2017)
	Colon cancer: DLD-1 cells	In combination with oxaliplatin, blocks cancer proliferation (synergic effect)	(Gazzerro et al., 2010)
	Breast cancer: MDA-MB-231	Inhibits cancer growth via a CB1R lipid raft/caveolae–mediated mechanism	(Sarnataro et al., 2006)
	Colon cancer: DLD-1, CaCo-2, and SW620 cells	Inhibits cancer growth, inducing mitotic catastrophe	(Santoro et al., 2009)
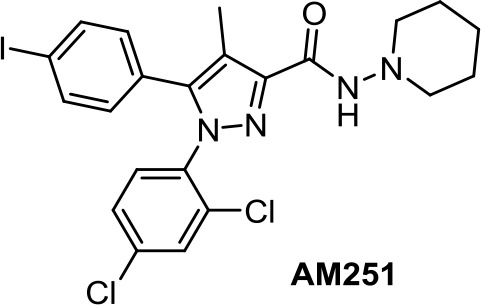	Breast cancer: MDA-MB-231	Increases invasiveness	(Mohammadpour et al., 2017)
	Lung cancer metastasis	Inhibits metastasis	(Marshall et al., 2011)
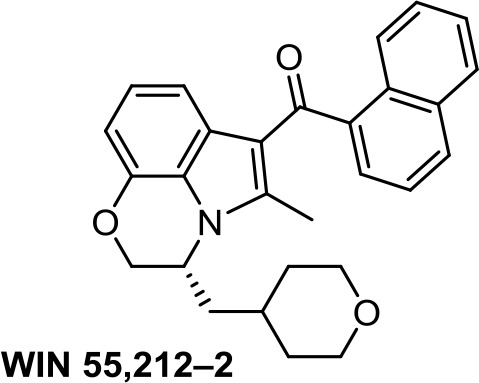	Renal carcinoma: 786-O, SMKT-R2, SMKT-R3, Caki-2, RCC-6, 769-P, Caki-1, and ACHN cells	Tumor growth inhibition and G0/G1 cell cycle arrest via CB2R activation	(Khan et al., 2018)
	Myeloma: U266, U266-LR7, RPMI, RPMI-LR5, MM1.S, and MM1.R cells	Proapoptotic effects	(Barbado et al., 2017)
	Lung cancer and testicular cancer: A549 and HoTu-10 cells	Proapoptotic effects	(Müller et al., 2017)
	Prostate cancer: LNCaP cells	Prevents neuroendocrine differentiation	(Morell et al., 2016)
	Gastric cancer: SGC7901 and AGS cells	Inhibits cell migration and invasion through COX-2 downregulation	(Xian et al., 2016)
	Hepatocellular carcinoma: BEL7402 cells	Induces cell cycle arrest and inhibits tumor proliferation and migration	(Xu et al., 2015)
	Breast cancer: MDA-MB-231, MDA-MB-231-luc, and MDA-MB-468	Inhibits tumor growth and metastasis	(Qamri et al., 2009)
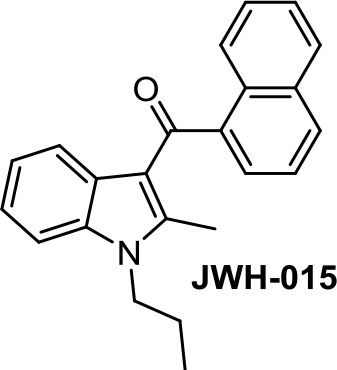	Breast cancer: 4T1 and MCF-7 cells	Apoptosis and reduction of metastasis	(Hanlon et al., 2016)
	Non–small cell lung cancer (NSCLC): A549 cells	Reduces tumor growth and inhibits macrophage recruitment	(Ravi et al., 2016)
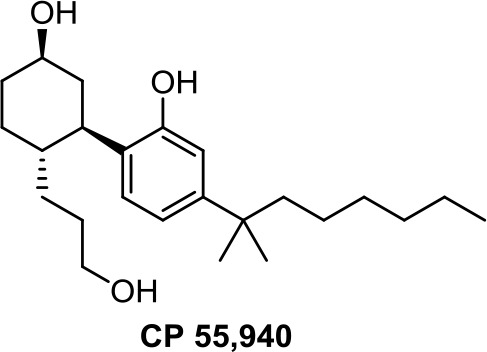	Gastric cancer: human AGS adenocarcinoma cells	Apoptosis induction	(Ortega et al., 2016)
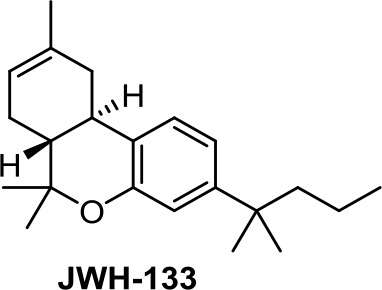	Breast cancer: MDA-MB-231, MDA-MB-231-luc, and MDA-MB-468	Inhibits tumor growth and metastasis via CB2R	(Qamri et al., 2009)
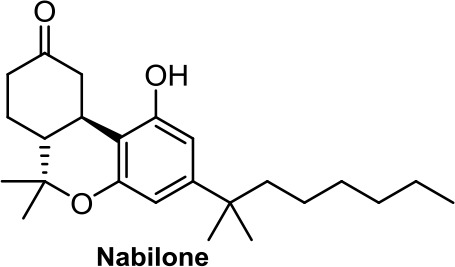	Human cancer patients	Chemotherapy-induced nausea and vomiting	(Velasco et al., 2016; Badowski, 2017)
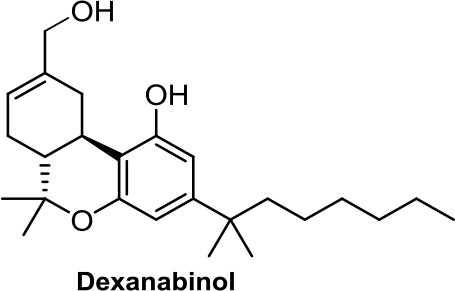	Brain cancer: human patients	Antiproliferative effects	(A Phase 1 Study of Dexanabinol in Patients With Advanced Solid Tumours, clinicaltrials.gov)
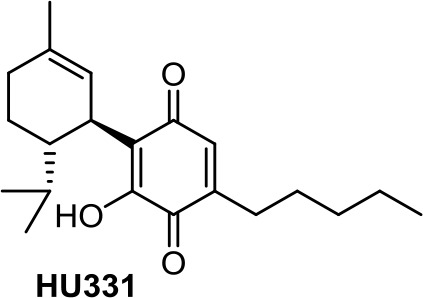	Leukemia, lymphoma, and colon cancer: Jurkat, Raji, and HT-29 cells	Inhibition of DNA topoisomerase II and antiangiogenic effects	(Kogan et al., 2004; Kogan et al., 2006; Kogan et al., 2007)
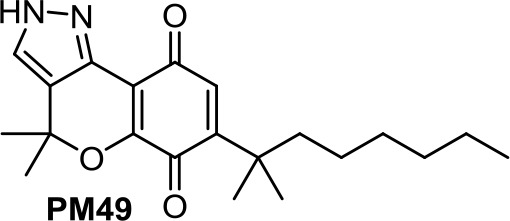	Prostate cancer: LNCaP and PC-3 cells	G0/G1 phase arrest and apoptosis through oxidative stress and activation of CB1R and PPARγ receptors	(Morales et al., 2013)
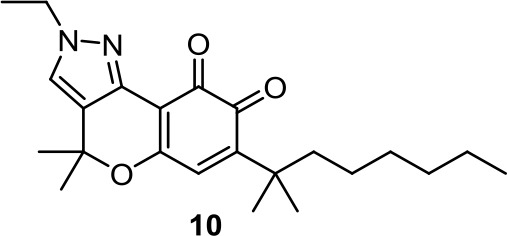	Breast cancer: MDA-MB-231 cells	Apoptosis through activation of CB2R receptors and oxidative stress	(Morales et al., 2015)
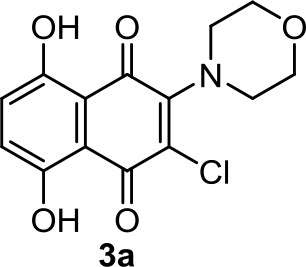	Breast cancer: MCF-10A cells	Antiproliferative effects related with its GPR55 activity	(Badolato et al., 2019)
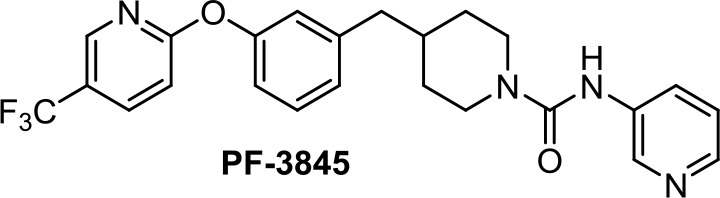	Colon cancer: Colo-205 cells	Reduces viability, migration, and invasiveness through FAAH inhibition	(Wasilewski et al., 2017)
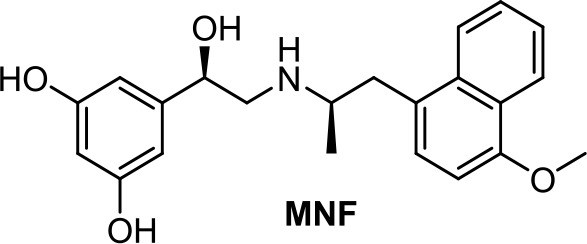	Hepatic and pancreatic cancers: HepG2 and PANC-1 cells	Impairs cancer cell motility via GPR55 signaling	(Paul et al., 2014)
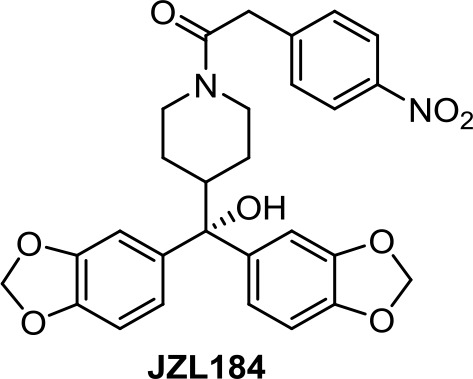	Colon cancer: HCT116, SW480, and LoVo cells	Regulates apoptosis and migration through MAGL inhibition	(Ma et al., 2016)
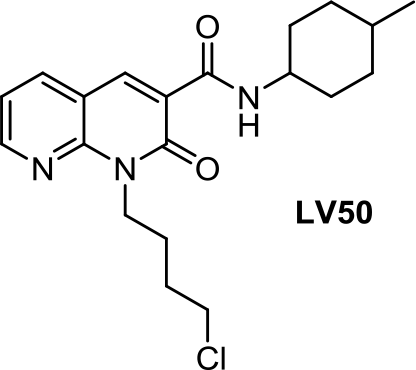	Leukemia: Jurkat cells	Antiproliferative and proapoptotic effect mediated through CB2R activation	(Capozzi et al., 2018)
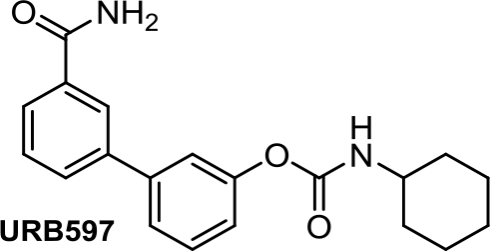	Lung cancer: A549 and H460 cells	Anti-invasive and antimetastatic action via FAAH inhibition	(Winkler et al., 2016)

Name/number from the original articles has been preserved.

Endogenous and phytogenic cannabinoids’ anticancer activity has been further reviewed elsewhere (Fidyt et al., 2016; Fraguas-Sánchez et al., 2016; Hinz and Ramer, 2019).

The anandamide synthetic analogue arachidonyl-2’-chloroethylamide (ACEA, **Table 1**) is a CB1R-selective compound, active in breast cancer stem cell invasiveness (Mohammadpour et al., 2017) but inactive in Kaposi’s sarcoma cells (Luca et al., 2009).

From a series of linolenic and arachidonic derivatives, **4g** and **5c** (**Table 1**) enhance AEA cytotoxicity on C6 glioma cell viability (Quintana et al., 2016). Both of them incorporate the same alkanolamine moiety in their structure.

### Phytocannabinoids

The plant-derived family is exemplified by the phytocannabinoids tetrahydrocannabinol [(-)-Δ^9^-THC); THC] and cannabidiol [(-)-CBD; CBD], the two main components of *Cannabis sativa*. Structural modifications of THC mainly have been developed in the Makriyannis laboratory some years ago (Thakur et al., 2005). Concerning CBD, a recent review dedicated to CBD as scaffold provides an overview of the chemical structure of natural and synthetic derivatives, including their relative molecular targets (Morales et al., 2017b). Reports on anticancer effects of phytocannabinoids mainly have been focused on the activity of Δ^9^-THC, CBD, Δ^9^-tetrahydrocannabinolic acid (Δ^9^-THCA), cannabidiolic acid (CBDA), cannabigerol (CBG), and cannabichromene (CBC) (**Table 1**) (Guzmán, 2003; Velasco et al., 2012; Fowler, 2015; Hinz and Ramer, 2019; Pellati et al., 2018). The effects of Δ^9^-THC have been tested in different cell lines of prostate cancer, breast cancer, colon cancer, pancreatic cancer, lymphoma, lung cancer, glioblastoma, and myeloma, among others (Fowler, 2015; Fraguas-Sánchez et al., 2016). Intracellular signaling through CBR has been shown to play an important role in these effects, involving complex signal transduction pathways, for instance, the ceramide pathway and/or the PI3-K and ERK pathways. However, the mechanism of action of Δ^9^-THC can also result in being CBR-independent, as has been shown for leukemic cell lines (Powles et al., 2005).

The nonpsychoactive cannabinoid CBD revealed proapoptotic effects in different cancer cell lines (Hinz and Ramer, 2019). CBR activation is not required for CBD anticancer action since CBD shows very low affinity. Accumulation of reactive oxygen species (ROS) is one of the main reported mechanism responsible for apoptosis induced by CBD (Ligresti, 2006). However, several molecular targets have been suggested, such as the COX-2, 5-LOX, PPARγ, mTOR, and p38 MAPK pathway (Hinz and Ramer, 2019).

The Δ^9^-THC plant precursor, Δ^9^-THCA, was shown to be slightly less active than its parent compound in human prostate carcinoma androgen receptor–negative and –positive cell lines (De Petrocellis et al., 2013), whereas in other cancer types such as breast, colon, gastric, glioma, and basophilic leukemia, they exert similar potency *in vitro* (Ligresti, 2006).

CBDA, the acidic precursor of CBD, inhibits the migration of MDA-MB-231 cells through COX-2 (Takeda et al., 2017), while CBC and CBG are much less active than CBD or inactive in different cancer cell lines (De Petrocellis et al., 2013).

Quercetin, a flavonoid present in fruits and vegetables, inhibits the growth of human colon adenocarcinoma cells through CB1R (Refolo et al., 2015). Another flavonoid structurally closely related to quercetin, morin (**Table 1**), showed an apoptotic effect by a mechanism not fully resolved (Hyun et al., 2015), but interestingly, morin also showed analgesic effects mediated through CB2R (Jiang et al., 2017).

Terpenes present in *Cannabis sativa* such as myrcene, α-pinene, and β-caryophyllene (BCP, **Table 1**) have been shown to exert synergic therapeutic actions with phytocannabinoids (Blasco-Benito et al., 2018). Anticancer and analgesic properties of β-caryophyllene have also been reported (Fidyt et al., 2016).

### Synthetic Cannabinoids

Medicinal chemistry programs focused on cannabinoids led to the discovery of different scaffolds that constitute the synthetic cannabinoid family (Vemuri and Makriyannis, 2015). In particular, CP-55,940, WIN55,212-2, JWH-015, JWH-133, SR141716 (rimonabant), SR144528, and ACEA have been considered excellent pharmacological tools to provide insights into the endocannabinoid system. The cyclohexylphenol CP-55,940, initially developed by Pfizer, was radiolabeled in Allyn Howlett’s laboratory (Yamada et al., 1996). Another CB1R/CB2R (cannabinoid receptor CB1/cannabinoid receptor CB2) mixed reference agonist is the aminoalkylindole WIN55,212-2 developed by Sterling Winthrop. From more than 400 cannabinoids synthesized in John W. Huffman’s laboratory, JWH-015 became a reference THC derivative for showing better affinity for CB2R than for CB1R (Huffman and Marriott, 2008). Then, with the naphthoylindole derivative JWH-133, Huffman’s team provided a potent selective CB2R receptor agonist versus CB1R.

#### Arylpyrazoles

Rimonabant (SR141716, **Table 1**), a CB1R inverse agonist, elicits alternative cell death pathways depending on the cell type affected. For example, Bifulco (Sarnataro et al., 2006) provides evidence for a lipid raft–mediated mechanism related to the CB1R in MDA-MB-231 cells, whereas it induces apoptosis in colon cancer through a CB1R-independent mechanism that involves the canonical Wnt/β-catenin pathway and β-catenin target genes (Santoro et al., 2009; Proto et al., 2017; Fiore et al., 2018). Rimonabant induces cell cycle arrest and programmed cell death in leukemia cell lines by a mechanism unlikely to be CB1R-dependent due to a low expression of CB1R in the cell lines used (U937 and Jurkat cells) (Gallotta et al., 2010). Recently, rimonabant has been shown to reduce colon cancer stem cell proliferation, which may account for cancer initiation, progression, and metastasis (Fiore et al., 2018). Synergy with antineoplastic drugs has been explored. A synergic antitumor effect was observed when combining rimonabant and oxaliplatin in colon cancer (Gazzerro et al., 2010).

AM-251 (**Table 1**) is a CB1R antagonist structurally closely related to rimonabant. It often has been used as pharmacological tool. For instance, AM-251 allowed determining of the functional relevance of CB1R signaling in Hodgkin lymphoma (Benz et al., 2013) and in rhabdomyosarcoma (Marshall et al., 2011). As an antitumor agent, AM-251 has not been reported to have significant differences with rimonabant.

#### Aminoalkylindoles

WIN55,212-2, a CB1R/CB2R dual agonist, has been one of the most widely used pharmacological tools to get insights into the endocannabinoid system. WIN55,212-2 (**Table 1**) inhibits cell proliferation and migration in triple-negative breast cancer (Qamri et al., 2009); in prostate cancer (Morell et al., 2016); in gastric cancer (Xian et al., 2016); in hepatocellular carcinoma (Xu et al., 2015); in lung cancer, testicular cancer, and neuroblastoma (Müller et al., 2017); in myeloma (Barbado et al., 2017); and in renal carcinoma (Khan et al., 2018). Most of these results were confirmed *in vivo* in various mouse model systems. The contribution of WIN55,212-2 to the proliferation relies on a different mechanism of action also involving cooperation processes. CB2R is clearly involved in hepatocellular carcinoma (Xu et al., 2015), myeloma (Barbado et al., 2017), and renal carcinoma (Khan et al., 2018), whereas both CB1R and CB2R contribute to the antiproliferative activity in triple-negative breast cancer (Qamri et al., 2009). In the case of prostate cancer (LNCaP), WIN55,212-2 preserves the levels of CB2R activity, which decrease during the neuroendocrine process (Morell et al., 2016). Cyclooxigenase-2 has been shown to be an important downstream target of WIN55,212-2 in gastric cancer metastasis (Xian et al., 2016). Moreover, this aminoalkylindole has been shown to improve inflammatory conditions, which ameliorate oncologic pathologies (Solbrig et al., 2010).

The naphthoylindole JWH-015 (**Table 1**) is characterized by high CB2R affinity, but it is not devoid of CB1R activity. CB2R activation has been reported to be involved in the antiproliferative effect of JWH-015 in different cancer cells, such as PC-3 prostate cancer cells (Olea-Herrero et al., 2009). In metastatic breast cancer MCF-7, crosstalk between CB2R and CXCR4 signaling seems to participate in the antiproliferative effect of JWH-015 (Nasser et al., 2011). In lung cancer cell lines, the effect of JWH-015 is comparable to WIN55,212-2, with CB1R/CB2R agonist-mediated antiproliferative effects (Preet et al., 2011). In cancer murine 4T1 and human MCF-7 mammary carcinoma cells, the action of JWH-015 seems to be complex, since it is not mediated either by CB1R or CB2R, or by GPR55, TRPV1, or TRPA1 receptors (Hanlon et al., 2016).

#### Quinones

Many quinones are cytotoxic through DNA intercalation, inhibition of DNA topoisomerase II enzyme, and free radical production. In this context, phytocannabinoids are interesting starting materials for preparing quinones. Thus, oxidation of CBD, ∆^8^-tetrahydrocannabinol, and cannabinol leads to *para*-quinone derivatives respectively named HU-331 (**Table 1**), HU-306, and HU-345 (Kogan et al., 2004). They all exert antiproliferative activity for Burkitt’s lymphoma, T-cell lymphoma, glioblastoma, breast cancer, prostate cancer, lung cancer, and colon cancer. Efforts have been focused on the mechanism of action of HU-331, whose antitumor effect has been shown as not being directly mediated by CB1R or CB2R receptors (Kogan et al., 2006). HU-331 was found to specifically be an inhibitor of topoisomerase II, while having no effect on topoisomerase I (Kogan et al., 2007).

Based on the synthetic cannabinoid scaffold chromenopyrazole, *para*- and *ortho*-quinones were reported (Morales et al., 2013; Morales et al., 2015). As indicated by their profile, CB1R/CB2R for *para*-quinones and CB2R for *ortho*-quinones, *para*-quinones (such as PM49, **Table 1**) inhibit prostate LNCaP cell viability through a mechanism involving oxidative stress, PPARγ, and partially CB1R (Morales et al., 2013), while *ortho*-quinones (such as **10**, **Table 1**) act on triple-negative breast cancer cells *via* CB2R activation and ROS production (Morales et al., 2015).

Recently, 1,4-naphthoquinone derivatives have been reported as efficient against triple-negative breast cancer (Badolato et al., 2019), which is not very surprising knowing that 1,4-naphthoquinone is a privilege scaffold for cytotoxicity. The cell viability assays assessed against the MDA-MB-231 cell line, which has been determined to overexpress GPR55 (Andradas et al., 2011), suggest that the most potent 1,4-naphthoquinone, 3a (**Table 1**), acts as an inverse agonist of GPR55.

#### Naphthyridine and Naphthalene

1,8-Naphthyridin-2-ones, CB2R agonists, have been shown to be, in general, more active against prostate carcinoma cells (DU-145 cell line) than MCF-7 breast carcinoma cells, gastric adenocarcinoma cells, and glioblastoma cells (Manera et al., 2012). Recently, the proapoptotic effect of the 2-oxo-1,8-naphthyridine-3-carboxamide LV50 (**Table 1**) on Jurkat leukemia cells was reported to be mediated by CB2R receptor (Capozzi et al., 2018).

Despite the expression of β2-adrenoceptor in the HepG2 hepatocarcinoma cell line and its β2-adrenergic properties, MNF [(R,R’)-4’-methoxy-1-naphthylfenoterol, **Table 1**] causes growth arrest and apoptosis through signaling pathways downstream of GPR55 rather than a β2-adrenergic–dependent mechanism (Paul et al., 2014).

#### Others

Apoptotic and necrotic cell death have been reported to be associated with elevated levels of AEA (Matas et al., 2007). Thus, inhibition of the enzymes involved in the biodegradation of the endocannabinoids has been shown to play a role in cancer cell viability, migration, and metastasis, as, for instance, does the FAAH inhibitor URB597 (**Table 1**) in lung cancer cells (Winkler et al., 2016). In colon cancer, FAAH inhibition (PF-3845, **Table 1**) seems to be a better strategy than MAGL (JZL184, **Table 1**) or DAGL inhibition (RHC-80267) (Wasilewski et al., 2017). However, in other studies, JZL184 was shown to have antiproliferative activity in apoptotic LoVo, HCT116, and SW480 cells (Ma et al., 2016).

The 2-(3-hydroxycyclohexyl)phenol CP-55,940 (**Table 1**), a well-known CB1R/CB2R agonist, has been used as a pharmacological tool for comparing the antineoplastic activity induced by endogenous and synthetic cannabinoids on gastric cancer cells (Ortega et al., 2016). CP-55,940 and AEA induce similar apoptotic effects, whereas Meth-AEA is more effective at inducing necrosis through transient and rapid apoptosis.

The benzo[*c*]chromene JWH-133 (**Table 1**), structurally related to ∆^8^-THC, has been chosen for studying the mechanism of action of synthetic nonpsychotic cannabinoids on breast cancer growth and metastasis due to its selectivity for CB2R (Qamri et al., 2009).

Interestingly, dexanabinol and nabilone (**Table 1**), synthetic analogues of THC, are the two synthetic molecules that have further progressed in the clinic. While nabilone is approved in certain countries for the treatment of chemotherapy-induced nausea and vomiting (Ware et al., 2008; Velasco et al., 2016; Badowski, 2017), dexanabinol is currently in clinical trials for the management of brain cancer (A Phase 1 Study of Dexanabinol in Patients With Advanced Solid Tumours, ClinicalTrials.gov; Dexanabinol in Patients With Brain Cancer, ClinicalTrials.gov). It is interesting to highlight that dexanabinol acts as an NMDA (N-methyl-D-aspartate) receptor antagonist and an inhibitor of the activity of nuclear factor kappa B (NF-kB) not binding CB1R and CB2R. Therefore, its antitumor molecular mechanisms could be mediated through the aforementioned targets (Striem et al., 1997; Jüttler et al., 2004).

Moreover, CB2R agonists have been the focus of molecular targets associated with photodynamic therapy (PDT) agents for developing target-specific PDT photosensitizers. In this sense, IR700DX-mbc94, a conjugate between a phthalocyanine dye and the CB2R inverse agonist SR144528, showed significant activity in the malignant astrocytoma cell line (Zhang et al., 2014). Another strategy is the co-administration of CB2R agonist and a PDT photosensitizer; synergic effects between the PDT agent IR700DX-6 T and JWH-133 have been observed in triple-negative breast cancer tumors (Zhang et al., 2018a).

Considering that GPR55 promotes cancer cell proliferation, peptide binders of GPR55 have been prepared and studied to inhibit the proliferation of EHEB and DeFew cells, two GPR55-positive B-lymphoblastoid cell lines (Mangini et al., 2017). These peptide binders are used as substitute tools for an antibody-based therapy strategy, since there is a lack of humanized monoclonal antibodies for this receptor.

## *In Silico* Admet Profile

Besides their activity and antiproliferative profile, pharmacokinetic aspects should be considered in selecting cannabinoid scaffolds for further development towards the oncology scenario. In this context, we have estimated the drug-likeness of the previously listed molecules.

ADMET properties were predicted using QikProp, integrated in the Maestro software (Schrödinger, LLC, New York, 2019) and the admetSAR web server (Cheng et al., 2012; Dong et al., 2018; Yang et al., 2018). Selected parameters are shown in **Table 2**. These calculations provide a common parameterization of physicochemical descriptors that allows comparison of ADMET profiles, which is a useful criterion for chemical probe selection for further development.

**Table 2:** Physicochemical descriptors of selected compounds as calculated using QikProp (integrated in Maestro, Schrödinger, LLC, New York, 2019) and the admetSAR web server (Cheng et al., 2012; Dong et al., 2018; Yang et al., 2018).

Compd	QPlogS^a^	QlogBB^b^	QPlogKhsa^c^	QPPCaco^d^	% Abs.^e^	hERG Blockage^f^	AMES Toxicity^g^	Carcinogenicity^h^	Acute oral toxicity^i^	LD_50_^j^	CYP substrate/inhibition^k^		
											CYP3A4	CYP2C9	CYP2D6
**AEA**	−6.20	−1.56	0.58	890	100	weak inhibitor	non-toxic	non-carcinogenic	III	1.52	substrate/non-inhibitor	non-substrate/non-inhibitor	substrate/non-inhibitor
**Met-AEA**	−5.02	−1.09	0.47	2,268	100	weak inhibitor	non-toxic	non-carcinogenic	III	1.65	substrate/non-inhibitor	non-substrate/non-inhibitor	substrate/non-inhibitor
**ACEA**	−4.72	−0.44	0.75	3,486	100	weak inhibitor	non-toxic	non-carcinogenic	III	2.24	substrate/non-inhibitor	non-substrate/non-inhibitor	substrate/non-inhibitor
**4g**	−6.18	−1.39	0.60	1,097	100	weak inhibitor	non-toxic	non-carcinogenic	III	1.99	substrate/non-inhibitor	non-substrate/non-inhibitor	substrate/non-inhibitor
**5c**	−5.18	−0.96	0.22	2,443	100	weak inhibitor	non-toxic	non-carcinogenic	III	1.43	substrate/non-inhibitor	non-substrate/non-inhibitor	substrate/non-inhibitor
**THC**	−6.64	−0.10	1.24	4,475	100	weak inhibitor	non-toxic	non-carcinogenic	III	2.59	substrate/non-inhibitor	substrate/inhibitor	non-substrate/non-inhibitor
**CBD**	−6.11	−0.49	1.06	2,437	100	weak inhibitor	non-toxic	non-carcinogenic	III	2.50	substrate/inhibitor	substrate/inhibitor	non-substrate/non-inhibitor
**CBG**	−6.19	−0.84	1.08	2,045	100	weak inhibitor	non-toxic	non-carcinogenic	III	2.29	substrate/inhibitor	non-substrate/inhibitor	non-substrate/non-inhibitor
**CBC**	−7.13	−0.42	1.29	3,569	100	weak inhibitor	non-toxic	non-carcinogenic	III	2.55	substrate/non-inhibitor	non-substrate/non-inhibitor	non-substrate/non-inhibitor
**Quercetin**	−2.89	−2.41	−0.34	18	52	weak inhibitor	non-toxic	non-carcinogenic	II	3.02	non-substrate/ inhibitor	non-substrate/non-inhibitor	non-substrate/non-inhibitor
**Morin**	−2.85	−2.34	−0.35	20	53	weak inhibitor	non-toxic	non-carcinogenic	II	3.08	non-substrate/ inhibitor	non-substrate/non-inhibitor	non-substrate/non-inhibitor
**BCP**	−6.22	1.04	0.96	9,906	100	weak inhibitor	non-toxic	non-carcinogenic	III	1.43	substrate/non-inhibitor	non-substrate/non-inhibitor	non-substrate/non-inhibitor
**SR141716**	−8.78	0.44	1.22	3,812	100	weak inhibitor	non-toxic	non-carcinogenic	III	2.54	substrate/non-inhibitor	non-substrate/inhibitor	non-substrate/inhibitor
**AM-251**	−9.02	0.47	1.27	3,812	100	weak inhibitor	non-toxic	non-carcinogenic	III	2.54	substrate/non-inhibitor	non-substrate/inhibitor	non-substrate/inhibitor
**WIN55,212-2**	−6.26	0.01	1.03	4,869	100	strong inhibitor	non-toxic	non-carcinogenic	III	2.47	substrate/inhibitor	non-substrate/non-inhibitor	substrate/non-inhibitor
**JWH-015**	−6.04	0.02	1.10	4,893	100	weak inhibitor	toxic	non-carcinogenic	III	2.52	substrate/non-inhibitor	non-substrate/non-inhibitor	substrate/non-inhibitor
**CP-55,940**	−6.49	−1.78	0.89	399	100	weak inhibitor	non-toxic	non-carcinogenic	III	2.08	substrate/inhibitor	non-substrate/non-inhibitor	non-substrate/non-inhibitor
**JWH-133**	−9.22	0.94	1.65	9,906	100	weak inhibitor	non-toxic	non-carcinogenic	III	2.13	substrate/non-inhibitor	non-substrate/non-inhibitor	non-substrate/non-inhibitor
**Nabilone**	−7.08	−0.81	1.24	1,348	100	weak inhibitor	non-toxic	non-carcinogenic	III	2.54	substrate/non-inhibitor	substrate/inhibitor	non-substrate/non-inhibitor
**Dexanabinol**	−7.25	−0.93	1.28	1,430	100	weak inhibitor	non-toxic	non-carcinogenic	III	2.51	substrate/non-inhibitor	non-substrate/non-inhibitor	non-substrate/non-inhibitor
**HU-331**	−5.35	−0.62	0.65	1,536	100	weak inhibitor	non-toxic	non-carcinogenic	III	2.34	substrate/non-inhibitor	non-substrate/non-inhibitor	non-substrate/non-inhibitor
**PM49**	−5.46	−1.19	0.45	507	96	weak inhibitor	non-toxic	non-carcinogenic	III	2.54	substrate/non-inhibitor	non-substrate/non-inhibitor	non-substrate/non-inhibitor
**10**	−6.27	−0.78	0.59	1,599	100	weak inhibitor	non-toxic	non-carcinogenic	III	2.65	substrate/non-inhibitor	non-substrate/non-inhibitor	non-substrate/non-inhibitor
**3a**	−1.96	−0.71	−0.66	342	78	strong inhibitor	non-toxic	non-carcinogenic	III	2.55	substrate/non-inhibitor	non-substrate/non-inhibitor	non-substrate/non-inhibitor
**PF-3845**	−7.26	−0.41	0.81	976	100	weak inhibitor	non-toxic	non-carcinogenic	III	2.83	substrate/inhibitor	non-substrate/inhibitor	non-substrate/non-inhibitor
**MNF**	−3.49	−1.21	0.20	91	79	weak inhibitor	non-toxic	non-carcinogenic	III	2.42	substrate/non-inhibitor	non-substrate/non-inhibitor	non-substrate/inhibitor
**JZL184**	−4.99	−1.40	0.30	217	77	weak inhibitor	toxic	non-carcinogenic	III	2.68	substrate/inhibitor	non-substrate/non-inhibitor	non-substrate/non-inhibitor
**LV50**	−6.41	−0.43	0.56	1,706	100	weak inhibitor	toxic	non-carcinogenic	II	2.65	substrate/inhibitor	non-substrate/non-inhibitor	non-substrate/non-inhibitor
**URB597**	−5.35	−1.31	0.34	321	89	weak inhibitor	non-toxic	non-carcinogenic	III	2.15	non-substrate/non-inhibitor	non-substrate/non-inhibitor	non-substrate/non-inhibitor

Physicochemical descriptors calculated by QikProp: ^a^Predicted aqueous solubility [-6.5/0.5]. ^b^Predicted log of the brain/blood partition coefficient [-3.0/1.2]. ^c^Prediction of binding to human serum albumin (-1.5–1.5). ^d^Apparent Caco-2 cell permeability [nm s^-1^, intestinal drug permeability, < 25 poor, > 500 excellent]. QikProp predictions are for non-active transport; ^e^Human oral absorption in the GI [< 25% is poor]; [range of 95% of drugs].

Toxicity parameters calculated with the admetSAR prediction tool: ^f^Predicted hERG blockade: compounds are classified according to the previously published approach as strong inhibitors (IC_50_ < 1 µM) or “non-blockers” exhibiting moderate (1–10 µM) and weak (IC_50_ > 10 µM) inhibitors (Marchese Robinson et al., 2011). ^g^AMES mutagenicity predictions are based on the previously published benchmark data set (Hansen et al., 2009; Xu et al., 2012). ^h^Carcinogenic potency is divided into three classes, labeled as “danger,” “warning,” and “non-required,” according to the TD_50_ (median toxic dose) values. Carcinogenic compounds with TD_50_ ≤ 10 mg/kg body wt/day were assigned as “danger,” those with TD_50_ > 10 mg/kg body wt/day were assigned as “warning,” and non-carcinogenic chemicals were assigned as “non-required” (Lagunin et al., 2009; Li et al., 2015).

^i^Compounds are classified into four categories based on the criterion of the US EPA (Category I contains compounds with LD_50_ values less than or equal to 50 mg/kg; Category II contains compounds with LD_50_ values greater than 50 mg/kg but less than 500 mg/kg; Category III includes compounds with LD_50_ values greater than 500 mg/kg but less than 5000 mg/kg; Category IV consists of compounds with LD_50_ values greater than 5000 mg/kg) (Li et al., 2014). ^j^Predicted median lethal dose (LD_50_) in rat model (acute toxicity in mol/kg) (Zhu et al., 2009).

^k^Metabolism parameters from admetSAR: Molecules were classified as substrate or non-substrate, and inhibitor or non-inhibitor of the different CYP450 isoforms, according to the previously published classification (Carbon-Mangels and Hutter, 2011; Cheng et al., 2011a, Cheng et al., 2011b).

According to our *in silico* calculations, most of the cannabinoids analyzed herein follow the Lipinski and Jorgensen pharmacokinetic rules (Lipinski, 2001; Jorgensen and Duffy, 2002). It is interesting to underline that our results are consistent with the experimental ADMET parameters published for some of these cannabinoids (Grotenhermen, 2003; Stout and Cimino, 2014; Zendulka et al., 2016). As shown in **Table 2**, the prediction of human oral absorption, blood–brain barrier permeability, bioavailability, human intestinal permeability, or binding to human serum albumin suggests that these cannabinoids have an appropriate drug profile. However, solubility as well as metabolic and toxicity parameters of specific compounds such as JZL184, LV50, JWH-015, or 3a fall outside the range predicted for FDA-approved small-molecule drugs (Hansen et al., 2009; Zhu et al., 2009; Xu et al., 2012; Li et al., 2014). Certain cannabinoids of phytogenic and synthetic nature may inhibit the activity of one or more cytochrome P450 isoforms (**Table 2**). Since these enzymes are involved in over 70% of human drug metabolism (Guengerich, 2008), their interactions with cannabinoids can affect drug clearance, consequently enhancing toxicity. This should be especially taken into account when combining these cannabinoids with other chemotherapy agents. Consequently, when moving forward toward the clinic, selected cannabinoids could be discarded for pharmacokinetic issues.

## Perspective

The first report on the antitumor activity of phytocannabinoids was published over four decades ago (Munson et al., 1975). Nevertheless, it is only in recent years that interest in these properties has grown. In addition to the well-established palliative effects of cannabinoids in cancer therapy, cannabinoids have attracted attention as possible anticancer drugs. There is a growing body of evidence showing that endogenous, phytogenic, and synthetic cannabinoids, and modulators of endocannabinoid biosynthesis, inhibit proliferation of a wide spectrum of tumor cells. In this report, we aim to provide a perspective of the current drug development scenario of cannabinoid-based antitumor strategies and their potential pathway to the clinic.

Endogenous cannabinoids and their synthetic derivatives have widely exhibited their ability to modulate cell proliferation, angiogenesis, and metastasis in a number of cancer cell types. However, concerning their possible exogenous application for cancer treatment, their lipid nature along with possible alterations of the metabolism of the endocannabinoid system may decrease the pharmaceutical interest for this family. In fact, eicosanoid degradation can trigger handling difficulties as well as chemical stability issues.

On the other hand, phytocannabinoids and their synthetic analogues are further moving toward the bedside as potential antitumor agents. The natural occurring scaffold has been the most explored for their antiproliferative potential. As previously summarized, compounds isolated from *Cannabis* such as THC or CBD can reduce tumor growth *in vitro* and *in vivo* through different mechanisms depending on the cancer type. Remarkably, pilot studies and early-phase clinical trials indicate positive results regarding the survival of glioblastoma patients upon treatment with combinations of the aforementioned phytocannabinoids (A Pilot Study of Dronabinol for Adult Patients With Primary Gliomas, ClinicalTrials.gov; TN-TC11G (THC+CBD) Combination With Temozolomide and Radiotherapy in Patients With Newly-Diagnosed Glioblastoma, ClinicalTrials.gov; Guzmán et al., 2006).

Besides its promising anticancer potential, the pharmacokinetics of CBD should be taken carefully in the oncological field. This compound can inhibit the cytochrome isoform CYP3A4 (Zendulka et al., 2016), which may alter the metabolism of other drugs when used in combination. This is especially relevant due to the current use of drug cocktails for cancer treatment.

In line with these findings, it is interesting to underline the promising cannabis entourage effect. Synergic antitumor responses have been observed upon cancer treatment with cannabis botanical preparations (Blasco-Benito et al., 2018), exhibiting better results than pure phytocannabinoids administrated separately. Therefore, cannabinoid combinations may provide an improved antiproliferative strategy for cancer management.

Due to intellectual property pharmaceutical aspects, synthetic derivatives of phytogenic cannabinoids such as dexanabinol are also at advanced preclinical stages for the potential treatment of solid tumors (A Phase 1 Study of Dexanabinol in Patients With Advanced Solid Tumours, ClinicalTrials.gov; Dexanabinol in Patients With Brain Cancer,” ClinicalTrials.gov).

Moreover, the cannabinoid–quinones analyzed herein also represent a promising chemotype for anticancer research. In addition to their multitarget antitumor actions, they present a suitable pharmacokinetic profile, being, in our opinion, a good drug-like prototype for further development. As commented, the putative cannabinoid receptor GPR55 is considered an emerging target in cancer therapy (Andradas et al., 2011). Thus, GPR55 antagonists should be explored as antitumor drugs. Moreover, due to the overexpression of this receptor in specific tumors, compounds that specifically bind GPR55 might represent valuable tools as tumor-targeting agents for delivery of classical chemotherapeutic drugs. In this regard, the previously mentioned 1,4-naphthoquinones, such as 3a, or naphthylfenoterols, such as MNF, could be interesting candidates for the pursuit of this cannabinoid anticancer approach.

Arylpyrazoles, aminoalkylindoles, and other cannabinoid derivatives such as napththyridine need additional antiproliferative *in vitro* and *in vivo* assays to be considered for further antitumor drug discovery stages. Endocannabinoid enzyme inhibitors such as URB597 and JZL184 should also be further explored since they may provide complementary anticancer strategies.

A major concern when considering new molecules as antitumor agents is their selectivity for cancer cells *versus* normal cells. Interestingly, phytocannabinoids such as ∆^9^-THC (Caffarel et al., 2006; Caffarel et al., 2008) and cannabinoid–quinones such as PM49 and 10 (Morales et al., 2013; Morales et al., 2015) have shown selective toxicity toward cancer cells *versus* their non-transformed counterparts. This should be taken into account when selecting suitable entities for further development.

The role of the endocannabinoid system in carcinogenesis is not fully unraveled. Therefore, it is difficult to choose a specific cannabinoid chemotype for optimal anticancer drug development. Nowadays, major hopes are coming from phytocannabinoids and their synthetic derivatives since they are steps forward in the clinic race. However, parallel systematic exploration of promising scaffolds presenting optimized ADMET profiles along with diverse mechanistic antiproliferative effects will probably provide wider antitumor spectra.

In this perspective, we have been reporting cannabinoid-based scaffolds as single anticancer agents. However, over the last years, new anticancer strategies toward clinical translation of cannabinoids have been explored. Combinational therapy involving synergies between cannabinoids and other anticancer agents is one of these approaches (Gazzerro et al., 2010; Torres et al., 2011; Scott et al., 2017; López-Valero et al., 2018; Zhang et al., 2018a). Such combined therapies allow targeting of tumor progression at different levels. Another strategy will be the use of cannabinoids in preventive conditions (Liu et al., 2010; Khan et al., 2018). Since inflammation is a common risk factor for cancer, and some cannabinoids have shown anti-inflammatory properties, they could play a role in chemoprevention.

## Data Availability Statement

All datasets generated for this study are included in the manuscript and/or the supplementary files.

## Author Contributions

The authors equally contributed to this manuscript.

## Funding

N.J. thanks the Spanish Ministry for its support through MINECO/FEDER SAF2015-68580-C2-2-R and RTI2018-095544-B-100. P.M. is grateful to the CAM program “Atracción de Talento,” number 2018-T2BMD-10819. We acknowledge support of the publication fee by the CSIC Open Access Publication Support Initiative through its Unit of Information Resources for Research (URICI).

## Conflict of Interest Statement

The authors declare that the research was conducted in the absence of any commercial or financial relationships that could be construed as a potential conflict of interest.
